# Probiotics as Potential Biological Immunomodulators in the Management of Oral Lichen Planus: What’s New?

**DOI:** 10.3390/ijms23073489

**Published:** 2022-03-23

**Authors:** Paola Zanetta, Margherita Ormelli, Angela Amoruso, Marco Pane, Barbara Azzimonti, Diletta Francesca Squarzanti

**Affiliations:** 1 Laboratory of Applied Microbiology, Department of Health Sciences (DiSS), Center for Translational Research on Allergic and Autoimmune Diseases (CAAD), School of Medicine, Università del Piemonte Orientale (UPO), Corso Trieste 15/A, 28100 Novara, Italy; paola.zanetta@med.uniupo.it (P.Z.); 20023176@studenti.uniupo.it (M.O.); 2 Probiotical Research Srl, Via Mattei 3, 28100 Novara, Italy; a.amoruso@probiotical.com (A.A.); m.pane@probiotical.com (M.P.)

**Keywords:** oral lichen planus (OLP), probiotics, apoptosis, cytokines, NF-kB signalling, T cells, immune-modulatory activity, microbial metabolites, host-probiotic interaction, microbiota

## Abstract

Oral lichen planus (OLP) is a T cell-mediated chronic inflammatory disorder with multifactorial aetiology and malignant transformation potential. Despite the treatments so far identified, new tailored and safe specific measures are needed. Recently, human microbiota imbalance has been linked to several immune-mediated diseases, opening new therapeutic perspectives for probiotics; besides their ability to directly interact with the host microbiota, they also display a strain-specific immune-modulatory effect. Thus, this non-systematic review aims to elucidate the molecular pathways underlying probiotic activity, mainly those of *Lactobacilli* and *Bifidobacteria* and their metabolites in OLP pathogenesis and malignant transformation, focusing on the most recent in vitro and in vivo research evidence. Findings related to their activity in other immune-mediated diseases are here included, suggesting a probiotic translational use in OLP. Probiotics show immune-modulatory and microbiota-balancing activities; they protect the host from pathogens, hamper an excessive effector T cell response, reduce nuclear factor-kappa B (NF-kB) signalling and basal keratinocytes abnormal apoptosis, shifting the mucosal response towards the production of anti-inflammatory cytokines, thus preventing uncontrolled damage. Therefore, probiotics could be a highly encouraging prevention and immunotherapeutic approach for a safer and more sustainable OLP management.

## 1. Introduction

Oral lichen planus (OLP) is a T cell-mediated chronic inflammatory disease of the oral mucosa, with an unclear multifactorial aetiology. Up to now, traumatic events, infections, diet, chemicals, and genetic susceptibility are identified as the main risk factors for OLP development [1,2,3]. It occurs in 0.5–4% of the global population, with a 1.5:1 female to male gender ratio [4]. This immune-related disorder comprises at least six different clinical patterns (reticular, atrophic, papular, bullous, plaque-like, and erosive-ulcerative types), whose lesions and symptoms may also affect other body sites and reduce patient quality of life [5,6].

Recently, human microbiota imbalance has been linked to the development of several immune-mediated diseases in anatomically distant sites [7]. In dysbiotic patients, opportunistic pathogens may over-colonize oral niches, in which they do normally reside, and express virulence factors causing the depletion of beneficial commensal bacteria and the onset of inflammatory features, also leading to cancer development [8,9]. One of these could be the typical band-like lymphocytic infiltrate, which represents the fingerprint of OLP disease and that is responsible for hydropic degeneration and destruction of the basal epithelial layer [6].

An updated review by Villa et al. investigated the possibility that the microbes themselves could cause the disease; however, the authors excluded this hypothesis. Opportunistic bacterial, viral, and fungal pathogens, that find a favourable environment for their growth and virulence expression, certainly contribute to the pathogenesis of the disease, not as triggers but rather as key cofactors, at least according to the current microbiological knowledge [10].

The first evidence of OLP malignant transformation was reported in 1910 [11], but only in 2005 was OLP declared by the World Health Organization (WHO) as a potentially malignant disorder [12]. It is still misrecognized due to the lack of specific and objective diagnostic criteria and, from recent studies, its progression ratio into oral squamous cell carcinoma (OSCC) ranges between 0.44% and 2.8% of the total OLP cases [13,14,15].

**Figure 1:**
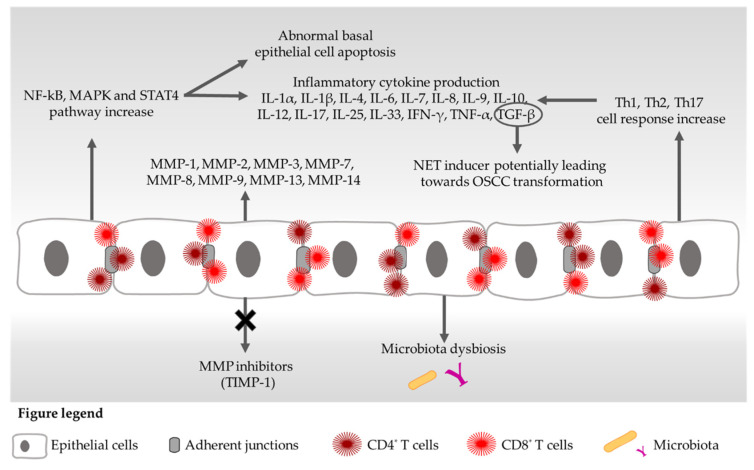
Overview of altered signalling pathways in OLP disease. IFN = interferon; IL = interleukin; MAPK = mitogen-activated protein kinase; MMP = metalloproteinase; NET = neutrophil extracellular trap; NF-kB = nuclear factor-kappa B; OSCC = oral squamous cell carcinoma; STAT = signal transducer and activator of transcription; TIMP = tissue inhibitors of metalloproteinases; TGF = transforming growth factor; Th = T helper cells; TNF = tumour necrosis factor.

OLP pathogenesis is strictly related to both specific and non-specific mechanisms [16,17,18]. The first one entails the activation of CD4^+^ and CD8^+^ cells after antigen presentation, which lead to the over-production of inflammatory cytokines, up-regulation of the nuclear factor-kappa B (NF-kB) signalling and abnormal apoptosis of the basal epithelial cells [17,18,19]. Moreover, the typical OLP immune dysregulation causes the production of mediators, not only by immune cells, such as macrophages, monocytes, CD4^+^, CD8^+^ and dendritic cells (DCs), but also from non-immune cells, such as fibroblasts, epithelial and endothelial cells [20,21]. On the contrary, unspecific mechanisms are controlled by matrix metalloproteinases (MMPs) and mast cell degranulation [17,18] (Figure 1).

OLP treatment mainly takes advantage of the use of corticosteroids, retinoids and 0.1% tacrolimus, especially for the control of the inflammatory symptoms [22,23,24]. Nevertheless, there is still the need to undertake more detailed, controlled, and comparative clinical studies to determine the balance between benefits and adverse effects after long-term therapy and to discover newly tailored, safer, and specific measures.

Up to now, many bacteria and fungi, such as *Lactobacilli*, *Bifidobacteria*, and *Saccharomyces* spp. respectively, have been exploited for human health since they display probiotic features and modulate the immune response in a strain-specific manner [25,26,27,28]. A proposed mechanism for the probiotic-mediated modulation of host immune cells concerns their capability to shift the balance of the mucosal response towards the production of anti-inflammatory cytokines [29]. Moreover, as depicted by Yu and Fan, they control and shape the T regulatory/T-helper 17 (Treg/Th17) axis, leading to the host protection from pathogens and hampering of an excessive effector T cell response [30,31]. Since probiotics dampen the interaction between T cells and antigens and suppress an excessive immune response by preventing uncontrolled damage and restoring the immune system homeostasis [29], they could be excellent candidates for OLP management at multiple levels.

This non-systematic review aims to elucidate the molecular pathways underlying the role of specific probiotic strains, mainly *Lactobacilli* and *Bifidobacteria* and their metabolites, in modulating OLP pathogenesis and malignant transformation, by focusing on the most recent research evidence. Findings connected to their activity in other inflammatory/immune-mediated diseases, that may suggest bacteria translational application in OLP, are here reported.

## 2. Cytokines and OLP

Cytokines are cellular mediators involved in both innate and adaptive immune responses. Aberrant production of these molecules can lead to immune deficiency, allergy and autoimmunity, as it occurs during OLP onset, relapse and tumoral progression [21].

Specific interleukin (IL)-1 superfamily members can be involved in OLP pathogenesis, such as IL-1α, IL-1β and IL-33, that were found overexpressed in OLP samples compared to normal oral mucosa [32,33,34,35]. Saliva displayed higher IL-1β levels compared to serum, with a positive correlation to the disease severity [36]; moreover, it was significantly increased also in the saliva of oral cancer-affected patients, suggesting its role as a potential OLP malignant transformation marker [37,38].

In a recent review, the authors reported that IL-4, whose production is also promoted by infections, has a key role in OLP development and may be used to develop novel preventive and therapeutic measures [39,40]. IL-4 was found at higher levels in both serum and saliva OLP samples compared to healthy controls, with a bigger difference in the serum [40,41]. In another in vitro assay, recombinant IL-25 expression, which correlates with both disease severity and IL-4 levels in specific OLP lesion subtypes, was used to stimulate patient-derived CD4^+^ T cells, leading to a significant IL-4 mRNA production increase [42].

Additionally, OLP-derived peripheral blood monocytes (PBMCs) and serum showed significantly higher IL-4 expression levels, but lower amounts of interferon-γ (IFN-γ) compared to controls [43]; this is also reflected in saliva samples, with a significant correlation between serum and saliva levels [44]. Conversely, the meta-analysis of Mozaffari et al. pointed out that no significant differences in IFN-γ levels were observed in OLP patient serum and saliva in comparison to controls [45]; however, several other studies proved that there is a strong IFN-γ presence in infiltrating lymphocytes in OLP patients’ saliva compared to controls [41,46,47]. Moreover, in an in vitro study in which IFN-γ was used to stimulate primary oral epithelial cells, the researchers demonstrated that this cytokine was able to induce epithelial to mesenchymal transition (EMT) and acquisition of the typical tumours stemness [48]. IFN-γ and IL-4, belonging to type 1 and 2 secreted cytokines respectively, were often investigated together, with IFN-γ/IL-4 ratio used as a marker of Th1 or Th2 response of the disease. While some authors found it significantly increased in OLP saliva samples compared to controls, indicating a predominance of Th1 response [47,49], other works showed that it decreased, thus displaying a predominance of Th2 response [44,50]. A recent meta-analysis did not evidence any difference in the IFN-γ/IL-4 ratio between serum and saliva samples of OLP patients and controls, probably due to the lack of standardization, especially for the saliva analysis [51].

NF-kB-dependent pro-inflammatory cytokines, such as IL-6, IL-8 and tumour necrosis factor-α (TNF-α), were also found to be involved in the pathogenesis of both oral premalignant and malignant forms [52,53,54]. In many studies and meta-analyses, they were highly expressed in OLP patient samples (mainly saliva and serum) compared to healthy controls [35,37,41,47,55,56,57,58]. The angiogenic process in OLP was increased by IL-6 [59], supporting its role in the pathogenesis of the disease [60] and, together with IL-8 and TNF-α, as a potential diagnostic and prognostic marker of malignant transformation [37,38,52,61]. While OLP saliva samples were more indicated for TNF-α and IL-6 monitoring [58,62,63], IL-8 levels were better detected in serum, being, in this case, a more sensitive marker than IL-6 to monitor the disease activity [64]. Moreover, IL-8 highly increased in OLP patient saliva when dysplasia was present and even more in oral cancer, thus also suggesting role as a malignant transformation biomarker [37,38,52]. In a recent study, Toader and colleagues demonstrated that IL-6 serum levels positively correlate with dyslipidemia, high triglycerides and the erosive and atrophic OLP clinical forms. As pointed out by the authors, these discoveries are moving the focus on OLP association with systemic inflammation and cardiovascular morbidity [65]. IL-6 increase could be due to the tripartite motif-containing protein 21 (TRIM21), which, if overexpressed in CD3^+^ T cells, causes T cell proliferation and IL-6 abnormal secretion via a tribbles homolog 2-mitogen-activated protein kinase (TRIB2-MAPK) signal axis [66,67]. Worthy of note, patients with both OLP and diabetes mellitus showed higher levels of IL-8 in serum and saliva compared to those with OLP only [68,69].

As for other cytokines, IL-10 was increased in OLP serum, saliva, and the infiltrating lymphocytes with respect to healthy controls [46,47,70]. Interestingly, mesenchymal stem cells (MSCs) from OLP lesions presented a higher expression of IL-6, IL-10, TNF-α and transforming growth factor-β (TGF-β) [71]. Like bacterial infections, TGF-β could induce the neutrophil extracellular traps (NETs) formation, which is associated with the potential OLP transformation into OSCC [72].

Other cytokines, such as IL-17 and IL-23 were found overexpressed in OLP lesions [46,73,74,75]. In vitro studies suggested that exogenous IL-23 could increase the percentage of Th17 cells and IL-17 production by CD4^+^ T cells of reticular OLP patients, and enhance the expression of cytokines, such as IL-8 and TNF-α, but not of IL-6 in human oral keratinocytes (HOKs) [73]. Renin, induced by NF-kB, also contributed to the increase of IL-17 in HOKs by enhancing signal transducer and activator of transcription (STAT)4 phosphorylation [76]. However, IL-17 seemed not to have a prominent role in OLP pathogenesis, since IL-17^+^ cells and IL-17 gene expression levels were not different among OLP samples and other OLP-unrelated inflammatory samples [77].

In a recent paper, Yang and colleagues investigated the role of T cell-derived exosomes in OLP pathogenesis, finding higher IL-7, IL-10, IL-12, and IL-17 and lower IL-1β, IL-5, and IFN-γ levels within this patient category compared to healthy controls and the ability to activate keratinocytes apoptosis in vitro [78].

Alongside all of this, new evidence is highlighting how cytokine levels are changing in OLP subtypes, also during or after treatments, allowing to better monitor OLP progression and response to therapy. For instance, serum IFN-γ was specifically increased in the erosive OLP group, while IL-4 was decreased [79]; importantly, other authors found that serum IL-4 was higher in patients with erythematous/ulcerative OLP compared to reticular one [44]. In saliva samples, instead, both IFN-γ and IL-4 were higher only in the erythematous/ulcerative OLP group compared to healthy controls, especially in saliva [80]. Moreover, the IFN-γ CD4^+^ T cell number was higher in patients with more aggressive erosive OLP forms [81]. The IFN-γ/IL-4 ratio, instead, did not display any difference among the different OLP subtypes [49]. IL-6 levels were consistent with OLP pathological features [66]; they were significantly higher in the saliva of atrophic-erosive and ulcerative forms compared to reticular ones [56,82], and, as for IL-10, they were positively and significantly correlated with reticulation/keratosis, erythema and ulceration scores [47]. Similarly, IL-8 was significantly higher in the saliva of erosive OLP patients compared to the reticular ones [53]. IL-23 and IL-17 expression positively correlated with reticular OLP tissues [73]. In the erosive OLP group, a higher number of IL-23 lymphocytes was present with respect to the reticular OLP group and inflammatory fibrous hyperplasia [83], while IL-17 serum levels were higher in erosive OLP patients compared to non-erosive OLP-affected subjects [84]. Among the treatments used to extinguish the cytokine storm, corticosteroids significantly reduced salivary IL-1β [36], levamisole modulated both IL-6 and IL-8 serum levels [64], topical dexamethasone decreased saliva TNF-α, IL-1α, IL-6 and IL-8 in erosive OLP patients by restoring IL-1α and IL-8 to the levels of the healthy controls [85], prednisone lowered IFN-γ and TNF-α in patients with erosive OLP [86] and, finally, total paeony glucoside improved the immunomodulatory function of MSCs by inhibiting IL-6 and TNF-α and increasing TGF-β and IL-10 expression [71].

Cytokine production shows multiple triggers. In in vitro experiments, it was proven how the Gram-negative lipopolysaccharide (LPS) enhanced IFN-γ and IL-1β production in HOKs in a hypoxia-inducible factor (HIF)-1α dependent manner, and how vitamin D interfered with this pathway by inactivating NF-kB [87]. Interestingly, Deng et al. demonstrated how patient salivary dysbiosis could correlate with toll-like receptor (TLR)4 and NF-kB p65 protein expression in OLP tissues and with IL-6 and TNF-α levels, consistent with those of NF-kB p65 [88]. Other authors observed that cytokine as IFN-γ and IL-33 levels were associated with OLP lesions, which also presented significant microbiota composition differences [89]; in fact, the bacterial community was less rich in erosive and reticular OLP patients than in healthy controls, with lower diversity in the erosive OLP group, and negatively correlated with salivary IL-17 concentration [90]. Wang and collaborators found that OLP fibroblasts significantly expressed more α-smooth muscle actin (SMA) compared to normal ones, indicating the presence of myofibroblast able to produce IL-6, IL-8 and TNF-α in response to *Porphyromonas gingivalis* LPS [91]. This bacterium, responsible for the onset of chronic periodontitis [92], when concurrent with OLP, caused a further increase of IL-17 levels [93,94]. This evidence highlights once more the role of the oral microbiota and that of the typical dysbiosis that may sustain the inflammation and the worsening of the OLP condition.

## 3. Matrix Metalloproteinases and OLP

Matrix metalloproteinases (MMPs) are a family of zinc-containing endopeptidases that mainly degrade connective tissue matrix proteins [16]. It was reported that the expression of different MMPs (MMP-2, MMP-9, MMP-14) increased progressively from normal oral mucosa to non-atrophic OLP, atrophic OLP and OSCC [95]. MMP-1 activation enhanced T cell accumulation in OLP, while MMP-9 inhibition prevented the collagen cleavage, resulting in the basal membrane integrity preservation [16]; moreover, these two MMPs, supported by bacterial plaque, were found highly expressed in patients with desquamative gingivitis secondary to OLP [96]. MMP-1 and MMP-9 levels were also found significantly higher in the gingival crevicular inflammatory exudate of OLP patients with chronic periodontitis and gingivitis, while the tissue inhibitor of metalloproteinase (TIMP-)1 was significantly lower than in chronic periodontitis patients without OLP [97].

Regarding MMP-9, it was only expressed in fibroblasts and the endothelium of small vessels in normal tissues [98], whereas in patients with oral premalignant disorders, as OLP, it was highly represented also in serum, saliva, lymphocytic inflammatory infiltrate of the lamina propria and basal and spinosum epithelial strata [99]. Moreover, MMP-9 mRNA positively correlated with IL-9 in OLP lesions and both were significantly elevated in the epithelial and lamina propria of erosive OLP compared to healthy controls; in addition, Th9/IL-9 could induce MMP-9 to aggravate OLP disease severity and increase IL-17 and Th17 cells [100].

Furthermore, MMP-2 was highly and mainly expressed in lymphocytic inflammatory infiltrate and lamina propria within the overlying epithelium; in particular, it was highly represented in atrophic/erosive than in reticular/papular OLP [101,102] and stimulated by LPS [103].

MMP-3 is crucial for cancer development and its serum and saliva levels in OLP patients progressively increased from reticular to erosive forms and further from low- to high-grade OSCC [104,105]. In support of this evidence, MMP-3, together with MMP-1, was found associated with erosion development [106]. Higher levels of MMP-7, MMP-8 and MMP-13 were also found significantly enhanced in OLP-affected patient serum and saliva [107,108,109].

## 4. Probiotic Effects on Cytokine/MMP-Mediated Signalling Pathways

The symbiotic relationship between human microbiota and host immune system is well established; when altered, it may contribute to the onset and progression of infectious and autoimmune diseases, whose inflammatory status and related symptoms seem to be ameliorated by probiotic treatments [110]. Regarding this, their role has been mainly investigated for chronic inflammatory diseases of the gastrointestinal tract, such as inflammatory bowel disease (IBD), ulcerative colitis (UC) and colitis-associated colon cancer.

In an IBD mouse model, *Bifidobacterium infantis* supplementation was able to increase Foxp3, IL-10 and TGF-β1 protein levels, besides alleviating intestinal epithelial injury and maintaining the intestinal immune tolerance [111]. *Lactobacillus acidophilus*, *L. casei*, *L. reuteri*, *B. bifidum* and *Streptococcus thermophilus* were demonstrated to be associated with CD4^+^Foxp3^+^ Treg up-regulation in inflamed regions, Th1, Th2 and Th17 cytokine downregulation and increased IL-10 and TGF-β expression levels in the IBD, atopic dermatitis (AD) and rheumatoid arthritis (RA) animal models [112]. In an infection-induced mouse model of colitis, *L. rhamnosus* ATCC 53103, *L. acidophilus* ATCC 4356 and *L. plantarum* A down-regulated the expression of IL-17, TNF-α and IFN-γ, and reduced the colitis-associated histological features [113]. In a chemical-induced mouse model of colitis, *L. fermentum* CQPC04 reduced TNF-α, IFN-γ, IL-1β, IL-6, and IL-12, and increased IL-10 release in serum; it also downregulated NF-kB and up-regulated IL-10 expression in colon tissue [114]. Extracellular vesicles from *L. rhamnosus* GG (LGG) prevented colon tissue damage and inhibition of TLR-4/NF-kB/NOD-, LRR- and pyrin domain-containing protein 3 (NLRP3) axis activation, resulting in the suppression of TNF-α, IL-1β, IL-2 and IL-6 expression [115]. *L. reuteri* I5007 significantly reduced IL-1β, IL-6, IL-17A, IFN-γ and TNF-α expression in colon tissue and increased IL-10 in the serum [116]. In patients affected by UC, *L. delbrueckii* and *L. fermentum* supplementation, together with sulfasalazine, was effective to ameliorate intestinal inflammation, resulting in a decrease in IL-6, TNF-α and NF-kB p65 concentration in the colon compared to patients treated with the drug alone [117]. In a colitis-associated colon cancer mouse model, *L. bulgaricus* decreased intestinal inflammation and reduced tumour levels of IL-1β, IL-6, IL-17, IL-23 and TNF-α [118]; *L. acidophilus*, *L. rhamnosus* and *B. bifidum* also reduced TNF-α and increased IL-10 in the colon of this mouse type [119]. *L. plantarum* YYC-3 cell-free supernatant (CFS) displayed an inhibitory effect on MMP-2 and MMP-9 production when used to treat colorectal cancer HT-29 and Caco-2 cell lines [120], while in human colorectal carcinoma HCT-116 cells, *L. casei* and LGG CFSs decreased MMP-9 levels, with *L. rhamnosus* GG displaying also an inhibitory activity on this MMP [121].

Inflammation is also triggered by infections, especially those carried by the LPS, which is used as a pro-inflammatory stimulus in several in vitro experiments. Chen and colleagues observed the ability of *L. delbrueckii* to significantly decrease TNF-α and IL-1β of LPS-challenged weaned piglets with respect to those treated with LPS alone; moreover, increased IL-10 levels in jejunum and ileum, as well as intestinal morphological changes indicating a better barrier function, were observed [122]. In RAW 264.7 murine macrophages challenged with LPS, a significant reduction of inflammatory markers, such as eicosanoids prostaglandin E (PGE)1, PGE2 and cyclooxygenase (COX) proteins, as well as the inhibition of IL-1β, IL-6 and TNF-α production, after *L. reuteri* LM1071 treatment was observed [123]. LPS was also used to induce inflammation in HT-29 cells, where *L. acidophilus* and *B. animalis* subsp. *lactis* reduced IL-8, phosphorylated NF-kB p65 (p-NF-kB p65), phosphorylated p38 MAPK, vascular cell adhesion molecule-1 (VCAM-1) and COX levels with an increase in TLR2 expression, showing a potent anti-inflammatory effect, by modulating the TLR2-mediated NF-kB and MAPK signalling pathways [124]. The same cells treated with *L. reuteri* I5007 before, together with, or after LPS, responded with significant cytokine levels production changes. In co-treated and post-treated groups, TNF-α and IL-10 levels did not modify compared to controls; conversely, the pre-treatment led to a significant up-regulation of the anti-inflammatory cytokine IL-10 and a significant decrease of the pro-inflammatory TNF-α and IL-1β [116]. The anti-inflammatory activity of *L. curvatus* MG5246 CFS towards human gingival fibroblasts treated with *P. gingivalis* LPS reduced PGE2 and COX2 and downregulated TNF-α, IL-6, MMP-2 and MMP-8 gene expression [125]. *L. paracasei* 06TCa19 suppressed IL-8 in human gastric epithelial cells MKN45 and AGS infected with *H. pylori* [126], while eleven *Lactobacillus* strains, isolated from Chinese fermented food, displayed the same effect on Caco-2 cells infected with *E. coli* [127]. *L. johnsonii* L531 reduced IL-1β, IL-6, IL-18 and TNF-α in *Salmonella typhimurium*-infected intestinal porcine enterocytes IPEC-J2 cells; moreover, it alleviated *S. typhimurium*-induced thigh junctions damage by inhibiting the TLR-4/NF-kB/NLRP3 inflammasome signalling pathway [128]. *L. rhamnosus* ATCC 7469 displayed its effects in enterotoxigenic *E. coli*-infected piglets, leading to a downregulation of IL-17A ileal expression, with no effects on IFN-γ, IL-4 and IL-12 in the small intestine, while the jejunal IL-2, ileal TGF-β and IL-10 were up-regulated in piglets receiving a lower dose of probiotics [129]. LGG oral administration in BALB/c mice, infected with *Giardia intestinalis*, enhanced the production of IL-10 and reduced pro-inflammatory IFN-γ [130].

The direct effect of probiotics on immune system cells was also studied. *L. acidophilus* and LGG CFSs significantly inhibited MMP-9 expression by THP-1 cells, increasing that of TIMP-1 [131]. *L. fermentum* IM12, isolated from the human gut microbiota, inhibited IL-6 expression and STAT3 activation in LPS-stimulated murine peritoneal macrophages [132]. PBMCs treated with *L. fermentum* KBL374 and KBL375, isolated from faeces of healthy Koreans, showed decreased levels of IL-2, IL-4, IL-13, IL-17A and IFN-γ, and an increase of IL-10 [133]. When *L. paracasei* SD1, *L. rhamnosus* SD11 and *L. rhamnosus* ATCC 53,103 were used to treat PBMCs, in combination with CFSs or cell wall extracts (CWEs) of eight different *Aggregatibacter actinomycetemcomitans* strains, a significant reduction in bacterial-induced toxicity and IL-1β secretion was observed. *L. paracasei* SD1 showed the best effect in reducing the cytotoxicity and release of IL-1β, IL-6, IL-8 and TNF-α from PBMCs [134]. *L. reuteri* CRL1098 soluble factors reduced TNF-α production by PBMCs and murine macrophages challenged with LPS, together with IL-6 in this last condition [135]. Jhun and collaborators showed that a combination of *L. acidophilus* LA-1, vitamin B and curcumin downregulated Th17 cells and IL-17, while the production of IL-10 was increased in human PBMCs isolated from osteoarthritis patients. In the mouse model of this disease was observed reduced pain and cartilage preservation; MMP-13 and pro-inflammatory cytokines IL-1β, IL-17 and TNF-α expression was decreased, while TIMPs were upregulated [136].

In other chronic inflammatory diseases such as osteoarthritis, or autoimmune diseases such as multiple sclerosis (MS), it was observed that the co-administration of *L. casei* with type II collagen and glucosamine to osteoarthritis-affected mice decreased the expression of the pro-inflammatory IL-1β, IL-2, IL-6, IL-12, IL-17, TNF-α, IFN-γ, and MMP-1, MMP-3, and MMP-13, and up-regulated the anti-inflammatory cytokines IL-4 and IL-10. Moreover, a reduced translocation of NF-kB into the nucleus and increased expression of the TIMP-1 in chondrocytes was observed [137]. An MS mouse model, with demyelination induced by cuprizone and treated with *L. casei* strain T2 IBRC-M10783, showed a significant decrease in IFN-γ and IL-4 serum levels compared to the cuprizone group alone [138].

Regarding oral pathologies, Han and colleagues showed how probiotics can interact with many pathways involved in OLP pathogenesis. Probiotics can modulate, in a strain-specific manner, immune response, microbial infection predisposition, T cell activation, infiltration and proliferation, keratinocyte apoptosis, NF-kB signalling, inflammatory cytokine production, MMP-9 expression and mast cell degranulation [17]. *L. reuteri* supplementation was useful in chronic periodontitis patients, whose gingival crevicular fluids showed a decrease in MMP-8 levels and an increase in TIMP-1 compared to the placebo group [139]. It was also observed that a 30-day supplementation with *L. acidophilus* BA05, *L. delbrueckii* subsp. *bulgaricus* BD08 (reclassified as *L. helveticus*), *L. paracasei* BP07, *L. plantarum* BP06, *B. longum* BL03, *B. infantis* BI04, *B. breve* BB02 (BL03 and BI04 reclassified as *B. animalis* subsp. *lactis*), and *S. thermophilus* BT01 induced a salivary IFN-γ reduction trend in symptomatic OLP patients, even though the difference was not significant with respect to the controls [140]. In an in vitro study, Li and collaborators observed that treatment with *Streptococcus salivarius* ATCC BAA-2593 supernatant of human squamous carcinoma HSC-3 cell line significantly reduced IL-6 expression; a decreased tendency was observed for IL-1β, IL-8 and TNF-α, showing an NF-kB pathway inhibition [22].

From all these studies, it is clear how probiotics are involved in the immune system response regulation, especially for what concerns inflammation. The major pathway involved in inflammation, and which probiotics can interact with, turned out to be that of NF-kB [27,141].

## 5. Probiotics and NF-kB Pathway: Evidence from Autoimmune Diseases and OLP

The p50 and p65 subunits of NF-kB are inhibited from entering the nucleus by IkB and related proteins. When pro-inflammatory cytokines, bacteria, viruses, reactive oxygen species and mitogens bind membrane and intracellular receptors, NF-kB is activated by the inducible degradation of IkB through the canonical pathway. When in the nucleus, NF-kB can promote the expression of several genes involved in cell survival, apoptosis, innate and adaptative immunity, lymphoid organogenesis, central and peripheral tolerance, thus increasing inflammatory cytokines, chemokines and adhesion molecules production and release [142].

Therefore, NF-kB could contribute to inflammation and/or autoimmunity, such as IBD (which includes UC and Crohn’s disease), atherosclerosis, MS, RA, type 1 diabetes, and OLP [143].

It has been demonstrated that *Bifidobacterium* strains may reduce inflammation. The recombinant *B. bifidum* BGN4, expressing superoxide dismutase and catalase, demonstrated in vitro antioxidant effects in H_2_O_2_-stimulated HT-29 intestinal epithelial cells and anti-inflammatory activities in LPS-stimulated HT-29 cells and RAW 264.7 murine macrophages [144]. The inhibitory effect of these *Bifidobacteria* on NF-kB seemed to be specific for LPS-induced inflammation in HT-29 cells in a dose- and strain-dependent manner [145]. *B. breve, B. longum* and *B. adolescentis* downregulated pro-inflammatory cytokines mRNA levels in LPS-stimulated RAW 264.7 cells [146]. The treatment with *L. plantarum* CAU1055 showed similar effects [147]. *B. breve* and *B. longum* Bif10 and Bif16 avoided gut microbial dysbiosis in murine models of colitis, in which *B. bifidum* showed high anti-inflammatory capacity [148,149].

Regarding *Lactobacillus* species, *L. reuteri* CRL1098 soluble factors modulated the inflammatory response triggered by LPS in RAW 264.7 by reducing the translocation of NF-kB p65 subunit from the cytosol into the nucleus, thus inactivating its pro-inflammatory transcription [135]. LGG treatment demonstrated significant amelioration of liver injury in caecal ligation and puncture-induced septic rat models (60 male 20–22-week-old Sprague-Dawley rats) by decreasing inflammatory cytokines mRNA and protein levels, including NF-kB [150]. Moreover, the soluble protein HM0539 from LGG modulated the TLR4/myeloid differentiation primary response 88 (Myd88)/NF-kB axis involved in the inflammatory response in IBD [151]. Rocha-Ramírez et al. demonstrated that human macrophages better fight pathogens like *Staphylococcus aureus*, *S. typhimurium* and *E. coli* when pretreated with four *Lactobacillus* strains (LGG, *L. rhamnosus* KLSD, *L. helveticus* IMAU70129 and *L. casei* IMAU60214), which increased the NF-kB p65 and TLR2-dependent signalling [152]. Another example is the use of *L. johnsonii* L531 which alleviated, through the NF-kB inflammasome pathway inhibition, the damage caused by *S. typhimurium*. Its infection was strongly correlated with the presence of NF-kB in the nuclei of IPEC-J2 cells, whereas most of the NF-kB p65 subunit was mainly found in the cytoplasm when cells were pre-incubated with the probiotic, thus demonstrating its function in hindering inflammation [128]. Moreover, the oral administration of heat-inactivated *L. fermentum* IM12 isolated from human gut microbiota significantly suppressed NF-kB activation and in vitro expression on LPS-stimulated mouse peritoneal macrophages, and in vivo on mouse models of carrageenan-induced hind-paw oedema or 2,4,6-trinitrobenzene sulfonic acid (TNBS)-induced colitis [132]. Similarly, the use of *L. delbrueckii* and *L. fermentum* for 8 weeks downregulated NF-kB p65 in UC-affected patients, preventing relapses [117]. Furthermore, exopolysaccharides (EPSs) from *L. paracasei* IJH-SONE68 improved inflammation in an allergy mice model of contact dermatitis and ameliorated colon atrophy, stool consistency and hematochezia in a UC mice model [153,154].

Combining both genera, *B. breve* CNCM I-4035, *L. paracasei* CNCM I-4034 and *L. rhamnosus* CNCM I-4036 orally administered showed anti-inflammatory effects in the intestine of Zucker-Leprfa/female rats, model of non-alcoholic fatty liver disease. While *B. breve* CNCM I-4035 and *L. rhamnosus* CNCM I-4036 showed no effects on phosphorylated Akt (p-Akt)/Akt, p-NF-kB/NF-kB ratio, on the contrary, *L. paracasei* CNCM I-4034 increased the ratio between p-Akt/Akt and NF-kB. All three of the probiotic strains decreased both pro-inflammatory macrophagic gene expression and leukocytic infiltration in the liver [155].

Moreover, *Saccharomyces boulardii*-derived soluble factor exhibited anti-inflammatory properties, since it inhibited NF-kB activation and IL-8 production in monocytes and intestinal epithelial cells [156].

Regarding NF-kB role in OLP, 60 patients showed a strong NF-kB nuclear presence in the erosive-atrophic with respect to the non-erosive group, thus reinforcing NF-kB role in the pathogenesis of this immune-mediated disease [157]. Similar results came from the study of Shi et al. [158]. These data were further confirmed in 14 atrophic-erosive and in 16 reticular cases which were immunohistochemically analysed for NF-kB p65 and TNF-α expression. NF-kB p65 staining was higher in the nuclei of basal and parabasal epithelial keratinocytes of OLP patients compared to normal oral mucosa biopsies, also correlating with TNF-α overexpression, suggesting a reciprocal positive regulation loop [159]. The high number of NF-kB positive epithelial cells was also associated with cytotoxic cell infiltration content [160]. In addition, the amount of NF-kB p65 and Foxp3^+^ Tregs was more abundant in OLP lesions of 40 Chinese patients with respect to controls [161]. In further 32 OLP cases, glucocorticoid receptor α mRNA and protein levels were decreased and significantly inversely correlated with NF-kB, showing a crosstalk between these molecules [162]. In oral lichenoid disease, an increased expression of NF-kB was revealed in the basal mucosal epithelial layer compared to control samples, supporting once more the evidence of the involvement of the innate inflammatory reactivity in this pathology [163].

In a recent work, the O-GlcNAcylation-induced NF-kB signalling was examined in relation to NLRP3 inflammasome, in which caspase 1 and IL-1β are involved. Higher and statistically significant levels of O-GlcNAcylation, NF-kB signalling molecules and NLRP3 inflammasome were found in OLP tissues with respect to healthy controls. Further analysis on the NLRP3 inflammasome will clarify its role in OLP pathogenesis, as for other immune-inflammatory diseases [164]. Regarding this, caspase 4 seemed able to activate the NLRP3 inflammasome through a non-canonical way in human cells [165].

Regarding microbial dysbiosis, Deng and coworkers analysed the oral microbiota of OLP patients, finding a decrease in *Derxia, Haemophilus* and *Pseudomonas* genera, without changes in the total composition with respect to the control group. They demonstrated a positive increased correlation in tissues between TLR4 and NF-kB p65 and suggested that the microbiota shifting could promote the inflammatory state trigger, which is at the basis of the onset and progression of the disease [88]. However, a significant reduction in the relative amount of *S. salivarius* was detectable in OLP patients. The treatment with *S. salivarius* K12 was efficacy in the reduction of symptoms in a cohort of OLP patients and the supernatant from *S. salivarius* ATCC BAA-2593 revealed its action in inhibiting the NF-kB pathway in HSC-3 cells, an inflammatory pathway study model [22]. Moreover, in vitro findings obtained from the co-culture of oral keratinocytes from OLP patients and *Candida albicans* revealed that the exposure to this fungus inhibits cellular apoptosis acting on TLR2/MyD88/NF-kB signalling pathway and may represent a new therapeutic target [19].

## 6. OLP Apoptotic Pathways and Probiotics

The apoptotic programmed cell death can be normally activated from cells in response to the presence of intracellular abnormalities, such as mutations or viral infections, or from extracellular signals, depending on the intrinsic or extrinsic pathway triggered [166]. The increased permeability of the mitochondrial membrane, which characterizes the intrinsic pathway, favours the release of pro-apoptotic molecules into the cytoplasm under the control of the B-cell lymphoma protein 2 (BCL-2) family, which comprises the anti-apoptotic Bcl-2 and Bcl-XL, and the cell death promoter Bax and Bak. On the other hand, the extrinsic pathway is governed by death ligands such as TNF-α and Fas ligand which, upon binding with specialized death receptors, results in the recruitment and activation of procaspase 8 into caspase 8. Both pathways converge into caspase protease family activation [166,167].

To clarify which apoptotic pathway was mainly involved in OLP progression, Mattila and coworkers evaluated the expression of caspases 2, 3, 8, 9, and 12 in 70 atrophic lesions. The authors revealed that caspases 2 and 12 are the most represented, likely due to the high rate of intracellular stress in epithelial cells, thus indicating a propensity for the intrinsic path [168].

The role of the apoptotic process in OLP-affected epithelial and lymphocytic infiltrate was analysed in 32 reticular and erosive cases and 20 healthy oral mucosa samples. The terminal deoxynucleotidyl transferase dUTP nick-end labelling (TUNEL) methodology, thanks to its ability to detect apoptotic DNA fragmentation, evidenced more intense apoptotic signals in the epithelia of the erosive OLP form which, conversely, were less strong in the inflammatory infiltrate. Moreover, lymphocytes were dominant within the inflammatory infiltrate, especially in erosive OLP with respect to the reticular type and the controls, thus suggesting that the different apoptotic level is at the basis of T cell persistence and OLP worsening [169]. This evidence was confirmed by another study, in which a series of 30 samples showed a statistically significant higher apoptotic index in keratinocytes and lower in lymphocytes with respect to those observed in control subjects, with caspase 8 and NF-kB p65 overexpression in both cell types of OLP cases [158]. Proliferation and apoptotic indexes were also investigated in PBMCs collected from atrophic-erosive OLP patients. The erosive form showed a higher proliferation rate than healthy controls, while the reticular type was the lowest one; the apoptotic rate was low in both cases, thus reinforcing the role of a T cell-mediated immune response [170].

Moreover, caspase 3 and Bax were investigated in a heterogeneous cohort of lichen planus (LP)-affected patients with the final aim to explain the clinical differences within variants. Increased caspase 3 levels were observed in OLP with respect to cutaneous LP, while an intense Bax expression was retrieved in OLP compared to the control group [171,172]. However, no differences in caspase 3 and Bax expression between atrophic-erosive and reticular OLP forms were observed [173,174].

The epithelial cells within OLP lesions resulted almost negative for the anti-apoptotic Bcl-2, while OSCC demonstrated a weak positivity for this marker [174]. These data were confirmed in another paper, in which the authors studied Bcl-2 expression in OLP and oral leucoplakia (OL) disorders. While OLP showed the lowest Bcl-2 expression, OL displayed the highest. Bcl-2 rate allowed to discriminate OLP from OL with sensitivity and specificity values of 58.6% and 99.32%, respectively [175]. Bcl-2 was also demonstrated to be enriched in the inflammatory cells of both OLP and OSCC, thus sustaining malignant cell survival and increasing the risk of developing new mutations [176].

In a recent in vitro analysis, caspase 1 was also identified as a possible key factor in OLP pathogenesis, since it was associated with the increased expression of the inflammatory TNF-α, IL-1β, IL-6 and IL-18, and positively correlated with the immune-related RAC2, CYBB and ARHGDIB [177].

In addition, soluble Fas ligand in reticular and atrophic-erosive OLP patient whole blood increased compared to control, correlating with an augmented TNF-α level [178]. Other important information came from the study of Neppelberg et al., who demonstrated the relevant expression of Fas ligand and its receptor in the inflammatory infiltrate, whereas the positivity in the epithelium was mainly in the basal rather than in the suprabasal layers. Moreover, apoptosis seemed to be related to the epithelium thickness [179]. To deepen this finding, 15 samples of reticular and 15 of erosive OLP were analysed to correlate the apoptotic degree with the thickness of the epithelial lesions. The erosive form showed a higher apoptosis rate with respect to the reticular one, with both forms characterized by more apoptotic cells and a thinner epithelium than healthy oral tissues [180].

The apoptotic process is also monitored by the tumour suppressor p53, which controls cell cycle progression and induces programmed cell death when DNA is damaged. In physiological conditions, p53 levels are counterbalanced by the mouse double minute-2 homolog (MDM2), which induces its proteasome-mediated degradation [181]. p53 increased in OLP, oral epithelial dysplasia and OSCC lesions with respect to normal healthy mucosa samples, thus emphasizing its role as OLP malignant progression marker [182,183,184,185].

Regarding the probiotic role in apoptosis, it was investigated in several studies in colorectal cancer (CRC) cells. Using faecal shotgun metagenomic sequencing, Sugimura and colleagues demonstrated that the culture supernatant of *L. gallinarum* significantly promoted CRC cell apoptosis in murine models (male and female ApcMin/+C57 black 6 mice), but not of normal epithelial cells, thus confirming its protective activity towards intestinal tumorigenesis [186]. Another evidence supporting the pro-apoptotic effect of probiotics came from extracellular vesicles of *L. paracasei* PC-H1, which was analysed in some CRC cell lines (HCT116 and SW1116 from human colon carcinoma, and SW620 from a lymph node of a 51-year-old Caucasian male). Induction in the apoptotic process was also in vivo demonstrated in 4-week-old female BALB/c nude mice via the PDK1/AKT/BCL-2 signalling pathway, by enhancing Bax expression, while dampening that of PDK1, Bcl-2 and AKT, involved in cell proliferation and apoptosis [187]. Starting from the evidence that *Saccharomyces cerevisiae* was depleted in faecal samples of CRC patients analysed by shotgun metagenomic sequencing, Li et al. demonstrated that in antibiotic-treated mice receiving this fungus by gavage, *Helicobacter* was decreased, while *Proteobacteria* and *Firmicutes* increased. Moreover, CRC cell lines HCT116 and DLD1 treated for 4 h with *S. cerevisiae*, but not with its metabolites, showed an enhanced apoptosis rate. Taken together, these results indicate that *S. cerevisiae* may be involved in CRC progression, by acting on cell apoptosis [188]. Different probiotic strains, including *S. boulardii* (Unique-28), *B. bifidum* (UBBB-55), *L. reuteri* (UBLRu-87), *L. plantarum* (UBLP-40), *L. fermentum* (UBLF-31), *L. salivarius* (UBLS-22), *Bacillus clausii* (UBBC-07), *Bacillus coagulans* (Unique-IS2) and *S. salivarius* (UBSS-01), were put in contact with HCT116 cells for 24 h. Cancer cell apoptosis was induced again, confirming the possible use of selected probiotics as coadjutants for CRC [189].

Moreover, the supernatant from *S. cerevisiae* var. *boulardii* was investigated for its anticancer activity, revealing the ability to induce human gastric and breast cancer cell apoptosis and decrease their viability; these findings open the scenario for this probiotic-derived supernatant as a complementary therapy for tumours handling [190,191].

Then, tyndallized *L. brevis* strains, more efficiently than *L. paracasei*, inhibited the growth of HT-29 human colon adenocarcinoma cells and induced apoptosis in a time-, dose- and strain-dependent fashion, by increasing Bax, caspases 3 and 9 mRNA expression levels [192]. 

Furthermore, the in vitro administration of *B. animalis* subsp. *lactis* EPSs to an intestinal porcine epithelial cell line reverted the effects caused by an enteropathogenic *E. coli* strain, reducing apoptosis through caspase 3 and 8 downregulation [193].

*L. reuteri* CRL1098 soluble factors were demonstrated to decrease the pro-apoptotic Bax levels and modulate the inflammatory response triggered by LPS in murine macrophagic cells, together with a reduction in TNF-α, IL-6, NO, COX-2 and Hsp70 production [135].

Finally, gut microbiota restoration, which was obtained through the probiotic *L. helveticus* R0052 and *B. longum* R0175 administration, produced beneficial effects on neurodegenerative diseases, by respectively up and downregulating Bcl-2 and Bax expression in the hippocampus of LPS-exposed rats [194].

## 7. Probiotics, T Cells and Autoimmunity

The human microbiota is a key factor for the immune homeostasis regulation; how it interacts with the immune system and how it can behave as a shield against infections, autoimmune, allergic and chronic inflammatory diseases, is still an open question that needs further investigation [110].

Activated T cells, exaggerated production of Th1 cytokines (such as IL-1, IL-8, IL-10, IL-12, TNF-α), and lymphocytic infiltration are peculiar characteristics of OLP [195,196].

Tregs, a T cell subpopulation, play a key role in the control of immune responses and the induction of peripheral tolerance by inhibiting T cell activation and secreting anti-inflammatory cytokines, such as TGF-β and IL-10 [197]. Some probiotics, like the *L. rhamnosus* and *L. delbrueckii* strains, modulate the immune response and induce Treg differentiation [198].

In the paper of Zheng et al., monocyte-derived dendritic cells (MoDCs), obtained from porcine PBMCs and stimulated with both *L. johnsonii* or its CFS, showed an upregulation of co-stimulatory and MHC class II molecules on their surface, able to trigger their maturation. Moreover, MoDCs stimulated by *L. johnsonii* strongly induced CD4^+^ T cell proliferation, and to a lesser extent in the presence of the CFS only. Moreover, the stimulation of MoDCs with *L. johnsonii* improved the Th1/Th2/Treg-type cell balance, whereas the same cells stimulated by *L. johnsonii*-CFS mainly directed T cells to Th2/Treg subset polarization [199].

A serine-threonine peptide cleaved from a protein secreted by *L. plantarum* showed to in vitro interact with intestinal DCs and stimulate the production of regulatory IL-10, thus demonstrating the immunoregulatory activity of bacteria-derived peptides and their dialogue with the host [200,201].

Γ-aminobutyric acid (GABA) receptors were recognized in a broad range of immune cells for their involvement in several pro-inflammatory cytokine downregulation. By treating a model of concanavalin A-stimulated mesenteric lymph node cells with CFS collected from GABA-producing *L. brevis* BGZLS10-17, the immunoregulatory mechanisms involved in the dampening of Th17 and Th1 cell response and the production of anti-inflammatory cytokines (IL-10 and TGF-β) by Foxp3^+^ Treg were clarified. The tested supernatants triggered the mRNAs expression of Foxp3, TGF-β and anti-inflammatory cytokine IL-10. The GABA effects were clearly due to the ATG5-mediated induction of the homeostatic autophagic process [202].

In a recent study, Manirarora and colleagues compared the impact of three different probiotic strains, *L. casei* B255, *L. reuteri* DSM 17,509 and *L. plantarum* LP299v on systemic lupus erythematosus (SLE) onset and progression. Among all, *L. casei* demonstrated its capacity on (NZBxNZW)F1 (BWF1) lupus-prone mice to induce Treg differentiation in vivo, thanks to the promotion of the expression of co-stimulatory molecules fundamental for antigen presentation [203]. In another study, the administration of *L. rhamnosus*, *L. delbrueckii* and prednisolone enhanced the activation of CD4^+^ CD25^+^ Foxp3^+^ Treg cells in the spleen of an SLE mice model [198].

An experimental C57Bl/6j mice model, immunosuppressed by antibiotics-induced microbiota depletion, was treated with antibiotics for 8 weeks and then with *E. coli*, *L. johnsonii* or faecal microbiota transplantation (FMT). *L. johnsonii* recolonization culminated in the highest CD4^+^ and CD8^+^ cell numbers in the mice small intestine and spleen. Moreover, FMT restored most efficiently the gut immune homeostasis, having a positive effect on the recapitulation of the balance between pro- and anti-inflammatory environment and increasing the frequency of regulatory T cells [204].

In another study, the ability of *L. reuteri* 5454 and *B. animalis* subsp. *lactis* 5764 in inducing Tregs was evaluated both *in vitro,* through DCs/CD4^+^ T cell co-culture, and in vivo in mouse models of TNBS-induced acute colitis and infected with *Citrobacter rodentium*. The study reported a strain-dependent effect of the two probiotics under investigation. On one hand, Lr 5454 was able to induce Tregs in DC/CD4^+^ T cell co-cultures but failed in promoting DCs maturation. In contrast, *B. animalis* subsp. *lactis* 5764 induced IL-17A and IL-22 secretion and promoted DCs maturation, while having a minor impact on Tregs in comparison to Lr 5454 [205].

*P. gingivalis* is one of the main pathogens involved in periodontitis and OLP [206,207]. Ultrasonicates of *P. gingivalis* (ATCC 33277) and LGG (CICC 6141) were used to stimulate CD4^+^ T cell cultures. Pathogen products promoted a Th17 pro-inflammatory phenotype and dampened the proportion of CD25^+^ Foxp3^+^ Tregs through the TLR4 signalling pathway; instead, probiotic ultrasonicates increased the proportion of Tregs through the TLR2 pathway and decreased the Th17 preponderance, restoring Th17/Treg balance and maintaining the immunomodulatory action of CD4^+^ T cells [207].

Fan et al. compared two strains of *L. casei* isolated from human faeces and their role in the prevention or attenuation of RA thanks to integrated cross-omic approaches. *L. casei* CCFM1074 promoted a reduction of arthritic symptoms while *L. casei* CCFM1075 did not, but both strains suppressed plasmatic IL-6 levels and the presence of Th17 cells. Moreover, *L. casei* CCFM1074 short-chain fatty acids (SCFAs) enhanced the proportion of Treg cells in mesenteric lymph nodes, demonstrating their pivotal role in alleviating collagen-induced arthritis in vivo [31].

Another study investigated the impact of a cocktail composed by *L. plantarum* MH-301, *B. animalis* subsp. *lactis* LPL-RH, LGG-18 and *L. acidophilus* on oral mucositis (OM) induced by concurrent chemo-radiotherapy for the treatment of nasopharyngeal cancer. The probiotic cocktail attenuated tissue damage in OM-affected rats, ameliorated tongue tissue apoptosis, and dampened the upregulation of TLR4/NF-kB pathway. Finally, it reduced OM severity through the regulation of microbiota dysbiosis and the enhancement of immunity [208].

The regulatory effect of probiotics in attenuating excessive inflammation and counterbalancing inflammatory Th cell response was confirmed at multiple levels. The feeding of experimental autoimmune encephalomyelitis (EAE) specific pathogen-free female mice models (6–8-week-old C57BL/6 mice; 6–8-week-old CD44KO mice; and TCR δ−/−mice) with *L. acidipiscis* increased the proportion of Tregs in the intestinal epithelium, suppressing the development of EAE and inhibiting at the same time the differentiation of CD4^+^ T cells toward Th1 and Th17 phenotype [209]. The probiotic cocktail VSL#3 (*B. longum*, *B. infantis*, *B. breve*, *L. acidophilus*, *L. paracasei*, *L. delbrueckii* subsp. *bulgaricus*, *L. plantarum* and *S. thermophilus*) protected non-obese diabetic (NOD) mice models from type 1 diabetes, dampening intestinal inflammation and restoring gut immune homeostasis through rebalancing T effector/T regulatory cells in the gut mucosa and pancreatic lymph nodes [210]. In another study, the oral administration of heat-killed *L. reuteri* alleviated collagen-induced RA in female DBA/1 J mice model and increased the frequency of Treg CD4^+^ IL-10^+^ cells in the draining lymph of joints [211]. IRT5 probiotic powder containing 1 × 10^11^ CFU/g of each strain (*L. casei*, *L. acidophilus*, *L. reuteri*, *B. bifidum* and *S. thermophilus*) strongly suppressed symptoms of experimentally-induced myasthenia gravis in 6–8-week-old Lewis female rats. Moreover, DCs isolated from treated rats significantly promoted the conversion of CD4^+^ T cells toward CD4^+^ Foxp3^+^ T regulatory cells [212].

## 8. Conclusions

Several pathways are dysregulated in OLP-affected patients. In particular, an unbalanced inflammatory cytokine production, T cell infiltration, NF-kB signalling pathway and apoptosis dysregulation are observed. In the last years, oral microbiota dysbiosis is also gaining importance as either a trigger or a supporter of OLP onset and development.

**Figure 2:**
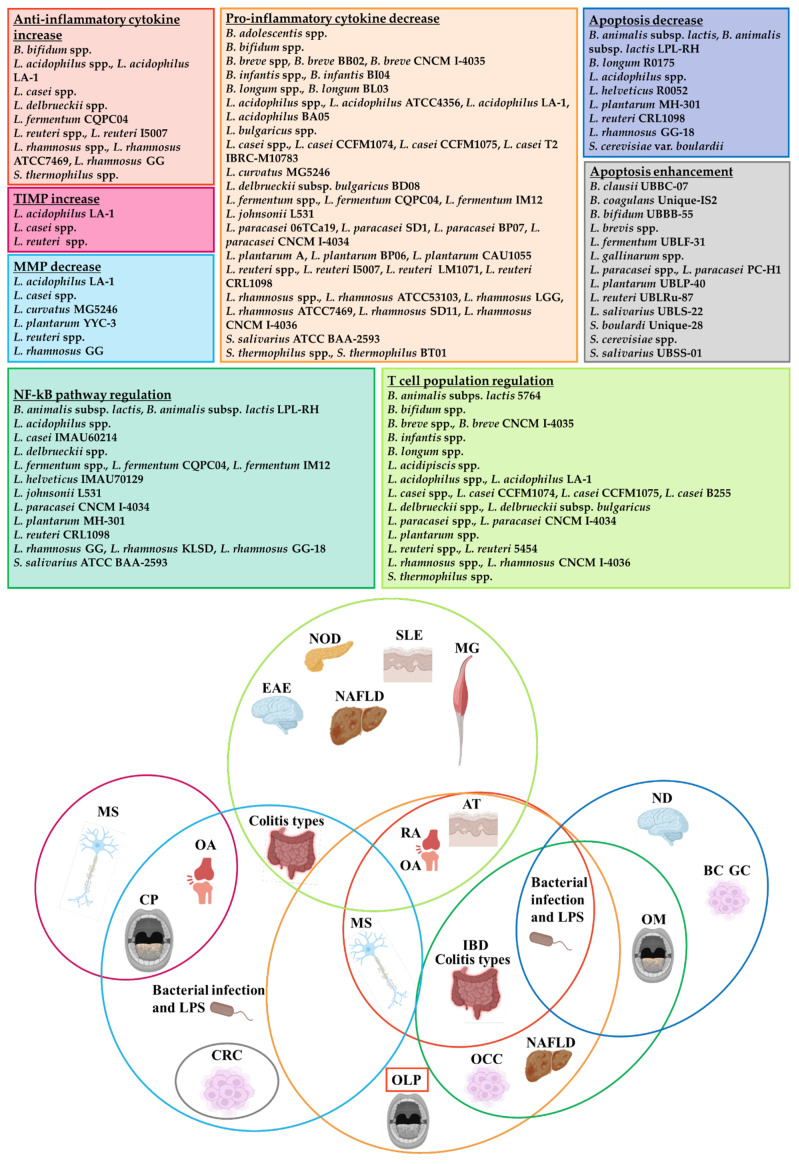
Impact of probiotics on inflammatory and apoptotic pathways and T cell population regulation. For each pathway, probiotics that display a regulatory effect are listed in the coloured boxes (upper part) and linked with the diseases in which this effect was observed (lower part), as evidenced by the circles with the same colour. AT = atopic dermatitis; BC = breast cancer; CP = chronic periodontitis; CRC = colorectal cancer; EAE = experimental autoimmune encephalomyelitis; GC = gastric cancer; IBD = inflammatory bowel disease; LPS = lipopolysaccharide; MG = myasthenia gravis; MMP = metalloproteinase; MS = multiple sclerosis; NAFLD = non-alcoholic fatty liver disease; ND = neurodegenerative disease; NOD = non-obese diabetic mouse; OA = osteoarthritis; OCC = oral cancer cells; OLP = oral lichen planus; OM = oral mucositis; RA = rheumatoid arthritis; SLE = systemic lupus erythematosus; TIMP = tissue inhibitors of metalloproteinases.

Since no specific treatments are available yet for the personalized management of OLP-affected patients, considering the microbiota involvement in the pathology, probiotic strains are noteworthy for novel therapeutic perspectives. The impact of probiotic administration is demonstrated to be beneficial without worsening pre-existent conditions. They modulate, in a strain-specific and time/dose-dependent manner, the microbial infection predisposition, inflammatory cytokines production, MMP-9 expression, NF-kB signalling pathways, keratinocytes apoptosis, mast cell degranulation and T cell activation, infiltration, proliferation and response (Figure 2).

Specific studies on the probiotic activity in OLP pathology are still missing, but the evidence available for other inflammatory disorders is underlying the potential benefits of their use also on this patient category. However, the authors of this review would like to express the need for more detailed, controlled and comparative long-term clinical trials to define personalized prevention and probiotic-based treatments.

## Data Availability

Not applicable.
